# Underfill: A Review of Reliability Improvement Methods in Electronics Production

**DOI:** 10.3390/polym17162206

**Published:** 2025-08-13

**Authors:** Zbyněk Plachý, Anna Pražanová, Karel Dušek, Attila Géczy

**Affiliations:** 1 Department of Electrotechnology, Faculty of Electrical Engineering, Czech Technical University in Prague, Technická 1902/2, 166 27 Prague, Czech Republic; 2 Department of Electronics Technology, Faculty of Electrical Engineering and Informatics, Budapest University of Technology and Economics, H-1111 Budapest, Hungary

**Keywords:** encapsulation, reliability, IC, PCB, underfill, flip chip, BGA

## Abstract

The increasing integration and miniaturization of electronic devices place serious pressure on packaging technologies to ensure long-term reliability. Polymer underfill encapsulation is a key process for reducing thermomechanical stress in modern assemblies. A systematic analysis that frames its diverse methods as solutions to the fundamental trade-off between the final polymer composite’s thermomechanical performance and its liquid-state processability is lacking from the literature. The novelty of this review lies in establishing a decision-making framework that connects specific application requirements to the underlying material science and process limitations. This article analyzes and compares different underfill techniques through a systematic literature review, from conventional capillary flow to advanced wafer-level underfills. Our findings show that this core trade-off leads to three distinct strategies: (1) Maximum reliability: This is achieved with highly filled, post-applied composites, offering excellent thermomechanical properties at the cost of slow, viscosity-driven manufacturing speeds. (2) High productivity: This is realized through integrated, pre-applied processes that simplify manufacturing but impose significant constraints on the polymer chemistry and filler content. (3) Targeted reinforcement for board-level packages: At the localized positions applied, ductile polymers often enhance mechanical shock resistance. This review concludes that the optimal underfill choice is not universal but is a complex, application-driven decision balancing the cured material’s performance against the processing demands of the polymer system.

## 1. Introduction

Electronics manufacturing is a complex discipline combining several process steps and production technologies. Electronics production does not cover only the assembly of the printed circuit board (PCB) with discrete components; it begins with the production of individual discrete elements and is followed by the assembly procedure. These process steps are generally known as electronic packaging, which can be defined as the science of integrating diverse materials, including semiconductors, polymers, ceramics, and metals, to perform critical functions such as signal and power distribution, thermal management to dissipate heat, and providing mechanical support and environmental protection for the integrated circuit (IC) [1,2,3,4].

The electronics packaging is structured in a hierarchical manner, from the zeroth level (the on-chip interconnections) through to the third level (board-to-board connections) and even beyond, with each level having unique material requirements. The applied materials must fulfill various conditions in terms of resistance to the external environment in the form of moisture, contamination, and heat dissipation, and they must ensure good electrical conductivity or insulation [4,5,6,7,8]. Moreover, considering current trends of producing smaller components or more complex ICs with a higher level of integration, the demands on these materials are crucial for the security of high device reliability. This is a challenge magnified by the inherent heterogeneity of the materials used, which, while essential for functionality, is a primary source of long-term reliability issues [1,9].

One of the most pervasive reliability challenges in electronic packaging is thermomechanical stress, the most common failure mode. Its root cause is the significant mismatch in the coefficient of thermal expansion (CTE) among the used materials. This issue is particularly significant in modern assemblies, where silicon chips are mounted on organic substrates like FR4. During thermal cycles in manufacturing and operation, this differential expansion and contraction subjects solder interconnects to severe cyclic strain, leading to low-cycle fatigue, crack initiation, and eventual failure. This problem is further intensified in advanced, high-density structures such as chiplet-based packages and 2.5/3D integrations. Adopting new wide-bandgap semiconductors like those made of silicon carbide for high-power applications introduces greater thermal loads, deepening the risk of thermomechanical failure. Beyond thermomechanical fatigue, delamination at the film/substrate interface is another critical failure mechanism that compromises the assembly’s structural integrity. Compounding these issues, the widespread use of chiplet technology in portable devices introduces a high susceptibility to failure from mechanical shock and drop impact [3,8,9,10,11,12,13].

Electronics production is challenging in terms of higher quality levels and long-term reliability [14,15,16,17]. In response to these challenges, especially for high-density interconnects like flip chips and BGAs, a diverse family of encapsulation technologies has been developed, with underfill being one of the most critical. Underfill is an engineered polymer composite, typically based on an epoxy resin matrix that is heavily filled with inorganic particles such as fused silica, with the filler content often reaching 70 wt.%. Applied as a liquid encapsulant into the gap between the chip and the substrate, its primary function after curing is to couple the two components mechanically. This rigid coupling redistributes the thermomechanical stress away from the fragile, individual solder joints and across the entire chip surface, thereby dramatically improving fatigue life and overall device reliability. The successful formulation of an underfill material involves achieving a delicate balance: its thermomechanical properties (a low CTE tailored to the solder, a high elastic modulus, and strong adhesion) must be optimized for reliability, while its rheological properties must allow for complete, void-free filling during manufacturing [4,8,18,19,20,21,22].

From a material science perspective, underfills are advanced composite systems where each component is engineered for a specific function. The polymer matrix, most commonly based on epoxy resins such as bisphenol-A, provides adhesion and determines the overall processing characteristics and chemical resistance. Other thermosetting polymers, such as cyanate esters, are also employed for specialized applications. The most critical component is an inorganic filler, typically fused silica, which is added in high concentrations to drastically reduce the composite’s CTE to match that of solder joints and increase its elasticity modulus. These filler particles’ size, morphology, surface chemistry, and dispersibility are crucial; they must be small enough to flow into micron-scale gaps without clogging yet large enough to achieve the desired mechanical reinforcement, creating a significant processing challenge. The formulation is refined with various additives, including curing agents, catalysts for controlling the reaction rate, adhesion promoters to enhance interfacial bonding, and toughening agents to prevent cracking [8,18].

The evolution of underfill technologies is intrinsically linked to the advancements in semiconductor packaging. While initially developed for flip chips on organic substrates, the proliferation of more complex architectures like 2.5 and 3D stacked ICs and chiplets has imposed new and stricter requirements on underfill materials. These advanced packages feature significantly a smaller standoff height, higher bump densities, and intricate geometries, demanding underfills with superior flow characteristics (rheology) and the ability to fill extremely narrow gaps without voids. Concurrently, the focus on reliability has expanded beyond just thermomechanical fatigue. Resilience against drop impact and mechanical shock has become a primary design driver for portable electronics. This had led to the development of alternative reinforcement strategies, such as corner and edge bonding, which often utilize more ductile, unfilled or lightly filled polymer systems. These materials are engineered to manage CTE mismatch and absorb and dissipate mechanical energy, representing a targeted material solution for different failure modes [4,8,9,10,19,20,21,22]. An overview of the typical thermomechanical properties for selected applied materials used in electronic packaging is presented in Table 1 to connect the basics with the future requirements of underfills.

To address the broad topic of underfills, our work provides a focused, comprehensive review of these distinct technologies. It analyzes their evolution, from solving the original problem of thermomechanical stress to meeting the modern demand for balancing maximum reliability with manufacturing efficiency. By comparing each method’s processes, materials, and key trade-offs between the thermomechanical properties of the polymer composites (driving reliability) and their processing characteristics like rheology (driving manufacturing efficiency), this paper aims to clarify their ideal application fields and provides a guide for selecting the optimal encapsulation strategy.

## 2. Review Methodology

This research is based on various sources, including scientific publications, professional monographs, popular science articles, and technical documentation from equipment and packaging material manufacturers. A systematic search was conducted in the Scopus and Web of Science databases using the names of individual techniques and related keywords. Relevant publications were subsequently selected from the results to create a comprehensive and coherent basis for this literature study. The analyzed works span the foundational concepts emerging in the mid-1960s to the latest publications from 2025. Although most sources come from the 21st century, older works are used mainly to place the issue in a broader context and describe the application areas of established technologies. At the same time, newer publications deal in detail with innovations in materials and process procedures. Following the manuscript’s drafting, Google Gemini was used to improve readability and the logical structure by identifying redundancies. The authors critically evaluated these suggestions and implemented appropriate revisions to finalize the text.

## 3. Results

**Figure 1:**
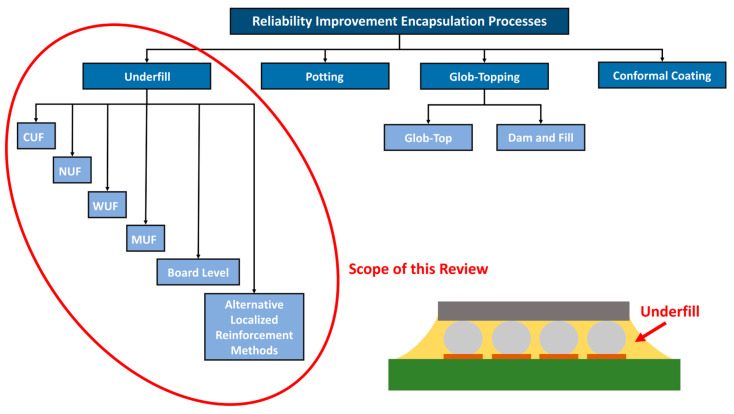
Distribution of reliability improvement encapsulation processes. The scope of this review is highlighted in red, focusing on underfill methods such as capillary underfill (CUF), no-flow underfill (NUF), wafer-level underfill (WUF), molded underfill (MUF), board-level underfill, and alternative localized reinforcement methods. The inset on the right provides a schematic cross-section of a flip-chip assembly, illustrating how the underfill material fills the gap between the chip and the base substrate.

The methods of underfill encapsulation processes were described individually in relevant subsections, covering conventional/capillary underfill (CUF), no-flow underfill (NUF), wafer-level underfill (WUF), molded underfill (MUF), board-level/second-level underfill, edge bonding, and corner bonding, as illustrated in Figure 1. Each subsection deals with the technology’s modifications, applications, advantages, disadvantages, and commonly used materials. In addition, this section comprehensively covers the given technologies, enabling their further comparison, application field discussion, and possible shortcomings and gaps that should be focused on in the future.

**Figure 2:**
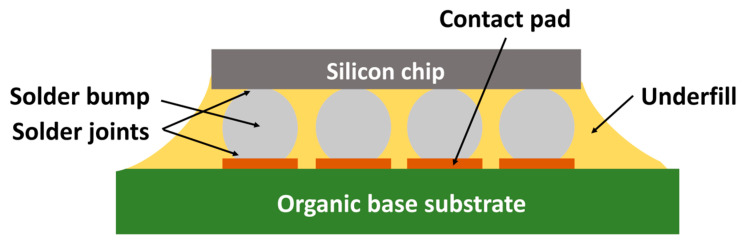
Illustration of C4 flip-chip packaging [5,7,26].

A significant breakthrough in electronics occurred in the 1960s when the American technology company IBM developed a novel packaging technology. This method involved creating solder bumps on the chip’s active side, which was then flipped over and directly soldered to the base substrate, giving rise to the name “flip-chip”. IBM named this innovative approach Controlled Collapsed Chip Connection (C4), enabling significant miniaturization and performance improvements in electronic components [14]. An illustration of the C4 flip chip is shown in Figure 2.

**Figure 3:**
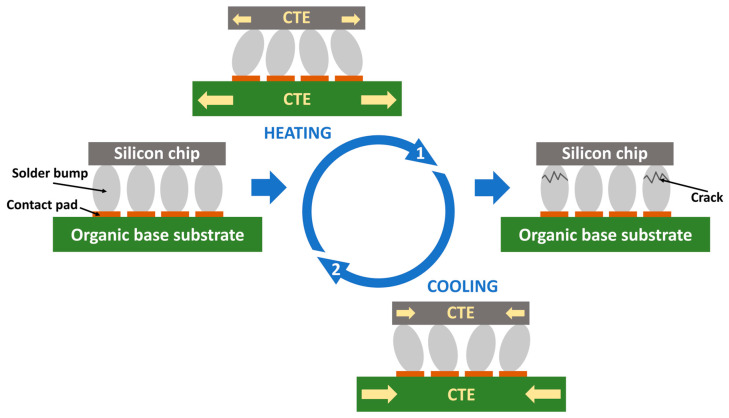
Schematic representation of the failure mechanism of a flip chip, which is driven by the coefficient of thermal expansion (CTE) mismatch between the chip and the organic substrate under thermal cycling [7,27].

From the beginning, chips were mounted on ceramic or silicon bases with a low coefficient of thermal expansion (CTE) matching that of the silicon chip. The problem with these substrates lies on the one hand in their high cost resulting from complex high-temperature processing and on the other hand in their high dielectric constant, which negatively affects signal delays [14,15]. As part of the effort to reduce the price and improve the transmission properties of flip chips, organic substrates, which are characterized by low costs and a low dielectric constant, began to be preferred as bases. The main problem with applying these substrates was their high CTE compared to the silicon chip. Typical CTE values for silicon (Si) are 2.5 ppm/°C, 4–10 ppm/°C for ceramics, and 18–24 ppm/°C for organic FR4. The difference between these values results from the thermomechanical stress of the solder joints during temperature cycling, leading to their cracking, as schematically shown in Figure 3. At the same time, as the distance from the neutral point increases, the stress on the soldered joints increases. Furthermore, the narrow gap between the chip and the base substrate leads to a high-stress concentration in solder joints. In other words, as the chip size increases, the magnitude of the shear stress caused by different CTEs also increases, and the reliability of the given assembly under thermal–mechanical stress becomes a critical issue [14,15,16,17].

A component created using flip-chip technology on an Al_2_O_3_ base, with resin filling the gap between the chip and the base, was introduced in 1987 by the Japanese technology company Hitachi. The purpose of the resin was to fill the space between the chip and the base substrate with a material with a CTE close to the solder joints. This approach became a technological demonstrator of improving the fatigue of soldered joints and later became known as “Underfill” [14,27].

Underfill is a liquid composite encapsulant consisting of a liquid epoxy resin matrix heavily filled with inorganic particles. After being applied in this liquid state, the material is cured at an elevated temperature, transforming it into a solid composite that provides the necessary thermomechanical reinforcement for the assembly. Currently, these particles are most often fused silica and other additives that enhance the overall properties of the resulting material. These properties include rheology, the modulus, the CTE, thermal conductivity, and more. After the underfill has been applied and cured, or after it has hardened, it is characterized by both a high modulus and a low CTE, corresponding to the CTE of solder joints, good adhesion to the chip and the base, and low moisture absorption. Subsequently, the underfill layer serves to redistribute the stress between the chip, the solder joints, and the substrate of the base instead of its concentration on the peripheral solder joints, thanks to which there is an overall increase in the service life of the solder joints or the entire system, as well as an increase in its overall reliability [5,14,15,16,17,27,28,29].

Currently, several underfill processes have been gradually developed, including CUF, NUF, WUF, MUF, board-level/second-level underfill, edge bonding, and corner bonding; they are used to a greater or lesser extent in their suitable application field.

### 3.1. Conventional/Capillary Underfill (CUF)

The terms “conventional underfill” and “capillary underfill”, both abbreviated as CUF, are commonly used interchangeably to describe the same process, in which a controlled amount of material is dispensed into the gap between the chip and the substrate to redistribute the thermomechanical stress caused by the different CTEs of the materials used [5,16,30]. The CUF is applied after the interconnection between the chip and the substrate is created. The underfill material is applied using a needle in a line along the adjacent edge of the chip. Subsequently, capillary action begins to wick the material under the chip, thus filling the space between the chip, substrate, and solder joints. The problem with this method is that the capillary flow is usually slow and incomplete, leading to voids and inhomogeneities within the underfill material [5,16,27,30].

#### 3.1.1. Process

**Figure 4:**
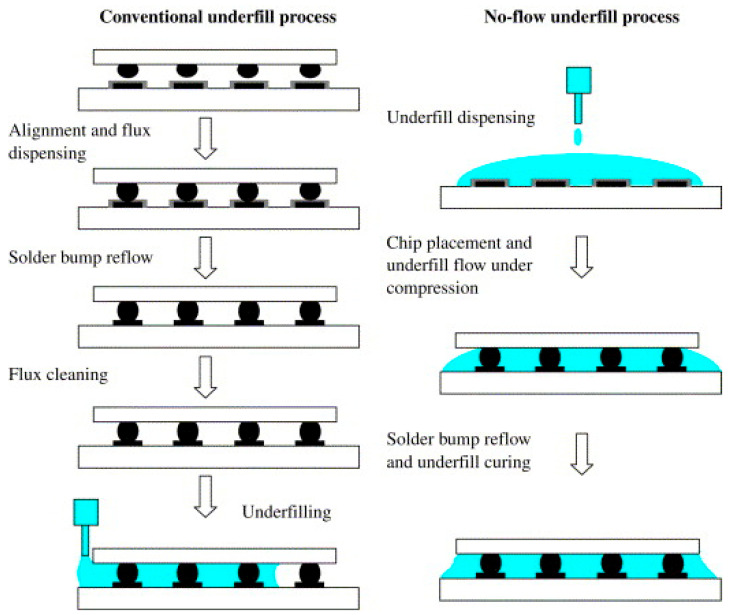
Conventional underfill processes vs. the no-flow underfill process [26] (reprinted from Microelectronics Journal, Vol. 38, Iss. 1, Wan, J.W. et al., Recent advances in modeling the underfill process in flip-chip packaging, pp. 67–75, © 2007, with permission from Elsevier).

To explain the process of applying CUF for flip chips and to understand the emergence of other methods, it is crucial to introduce simplified process steps of flip-chip production. In the first step, flux, a chemical agent designed to remove oxides from metal surfaces to ensure proper soldering, is applied to the substrate, typically using methods like precision dispensing. Subsequently, the chip, already prepared with bumps, is mounted on the substrate. The following step covers the creation of solid solder joints, which are realized using reflow soldering. The last step in this production is cleaning flux residues after soldering. Underfill is then applied to the flip-chip assembly, which is created and cleaned using dispensing systems, mainly by needle dispensing. This whole process is schematically shown in Figure 4. Underfill is applied just along the adjacent edge of the chip. The adhesive then flows under the component using capillary forces and fills the free space between the chip and the substrate. To facilitate a faster and more uniform capillary flow, the assembly is often preheated to a moderately elevated temperature, typically in the range of 70–100 °C. However, for snap-curable materials, this preheating temperature must remain below the onset temperature of accelerated curing to prevent the material from gelling prematurely before it can fill the gap completely. This preheating step significantly reduces the underfill’s viscosity. After the gap is completely filled, the final curing process takes place in an oven, where currently used adhesives are cured at temperatures up to 160 °C to achieve their final mechanical properties [5,17,27,31].

As the underfill flow became a bottleneck in flip-chip manufacturing, more attention was paid to it. In 1996, an equation describing the viscous flow between parallel plates driven by capillary forces was derived, allowing it to determine the underfill flow, which was introduced by Schwiebert et al. [32]. The flow time *t* (s) can then be defined as follows [32]:

(1) $$t\;=\frac{3\mu L^{2}}{h\gamma \cos\theta },$$

where *L* corresponds to the flow distance (m), *h* is the separation distance (m), *θ* is the wetting angle (°), *γ* is surface tension (N/m), and *μ* is the absolute viscosity (Pa·s). This equation neglects surface roughness, the effect of solder joints, and other obstacles. Moreover, in the work by Schwiebert et al. [32], the underfill flow rate dependence on its viscosity and surface tension was described. This work also determined the influence of hydrostatic pressure caused by gravity for the then-standard parameters of flip chips and adhesive properties to be roughly 1/5 of the capillary pressure. Therefore, better underfill flow improvement methods, compared to inclined or vertical flow, were discussed, leading to vacuum enhancement dispensing [32].

To speed up the application process, several experiments with dispensing patterns took place. One of the first was determining the flow-out time (s) effect of an “I” pass and an “L” pass introduced in the work by Babiarz et al. [33]. The mutual relationship between the given application patterns can be defined as [33]

(2) $$t_{I-line}=\sqrt{2}t_{L-shape}$$

**Figure 5:**
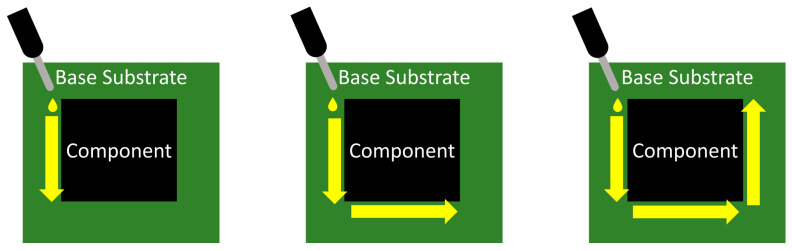
Schematic illustration of common dispensing patterns for the CUF process (from left to right: the I pattern, the L pattern, and the U pattern).

The work by Babiarz et al. [33] also pointed out that using other application patterns that will shorten the flow time compared to the classic “I” pattern or the “L”-shape pattern is possible. However, the other papers, for example, those by Abas et al. [34,35] and Ng et al. [36], focused on the underfill flow and the influence of dispensing patterns on underfill flow and void formation; based on the results, the “U” pattern with a shorter flow time than the “L” and “I” patterns is more susceptible to void formation. Moreover, the “I” pattern is more sensitive to incomplete filling [35]. All the mentioned works dealt with the influence of the solder joints, where the increasing number of soldered joints increases the flow time. A schematic representation of dispensing patterns is shown in Figure 5.

The formation of voids is a critical reliability concern in the CUF process, as their presence is a common cause of premature thermal cycle failure. These defects can act as initiation points for cracking and delamination and may even lead to electrical bridging between adjacent solder joints that are no longer fully encapsulated [20].

**Figure 6:**
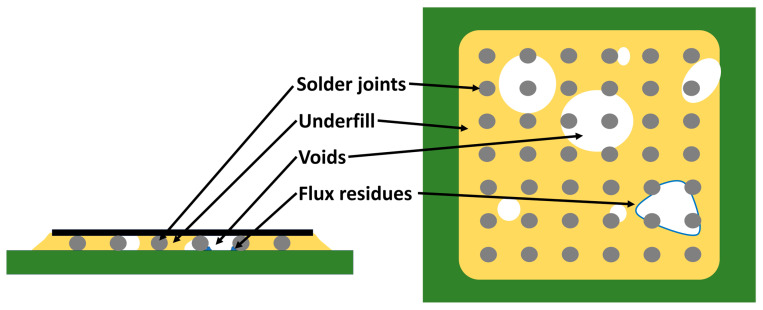
Schematic illustration of underfill voids. The diagram shows how contaminations, such as flux residues, can lead to the formation of voids between the solder joints.

Research by Ho et al. [37], Wang [38], De Sousa et al. [39], and Wakeel et al. [40] discussed in detail the influence of various factors on void formation in underfill, such as substrate surface roughness, plasma cleaning, the amount and compatibility of flux residues, and the size of fillers in the underfill. Wang [38] further discussed the rheological properties of fluxes and their influence on substrate wettability and subsequent underfill flow. De Sousa et al. [39] emphasized the importance of the preheating step before applying the underfill as a critical point for reducing void formation, as well as the uniform temperature of the parts during dispensing and the selection of an appropriate dispensing pattern. A paper by Chhanda et al. [41] investigated the influence of moisture exposure on the underfill’s mechanical properties and modeled the material’s viscoelastic behavior concerning moisture. Ying et al. [42] in their work showed that two factors are key for the voidless underfill process, one of which is the gap between the active surface of the flip chip and the solderless PCB mask, while the second factor is the PCB dwell time before the underfill process, where a shorter time reduces the risk of moisture-related voids. This work also shows that anhydride-based underfill has a higher capability to wash flux residues than phenol-based underfill. Thus, even with a suitable choice of underfill, the number of voids can be reduced, and a homogeneous underfill flow can be ensured. The common denominator of these articles [37,38,39,40,41,42] is the identification of the main causes of voids in the underfill, which are mainly flux residues, moisture, and the underfill dispensing process itself. The formation of voids from such contaminants is shown in Figure 6.

In addition to voiding, assembly warpage is another critical reliability factor arising from dispensing and curing. Warpage is a phenomenon primarily caused by the mismatch of CTE between the chip and the substrate. When the assembly heats up or cools down, its individual materials expand or contract to different extents, which leads to internal stress and subsequent deformation of the entire assembly [43,44]. Research by Zhang et al. [43] demonstrated that warpage is predominantly caused by the underfill due to CTE mismatch rather than by the solder joints, and it primarily develops during the cooling stage after curing [44]. While Fan et al. [45] confirmed that underfill increases overall package warpage, they also found that it beneficially reduces and redistributes stress on the fragile solder joints, which is the primary purpose of its application. This warpage primarily develops during the cooling stage after curing [44]. The assembly’s thermal history is also critical. According to Zhang et al. [43], the curing temperature defines the stress-free state, but if the assembly is heated above the glass transition temperature (*T*_g_), the *T*_g_ becomes the new stress-free reference. This shift can double the warpage, resulting in stress at room temperature. From a material engineering standpoint, warpage can be mitigated by adding toughening agents [44]. To achieve low warpage and high reliability, Sawada et al. [46] suggested designing underfills with extremely low elastic modulus and creep properties to absorb thermomechanical stress effectively.

The development of technology and underfill applications has also transitioned from needle dispensing systems to non-contact jetting dispensing systems. This change significantly improved the process by eliminating problems associated with the use of a physical needle. One major issue with needle dispensing is the risk of die chipping, which is the mechanical damage of the fragile silicon die’s edge or corner. This can occur if the needle makes physical contact with the die due to imprecise axis control or minor variations in assembly dimensions. The importance of a precise dispensing process is fundamental to reliability, as it ensures that the correct volume of material is consistently delivered to a specific location to achieve a complete, void-free underfill and form optimal filets. Jetting technology offers these advantages, enabling the creation of smaller filets, reducing the requirements for height measurements, and minimizing the volume variation from die to die that was inherent to needle dispensing. This is realized by dispensing the underfill using small, precisely controlled drops [47,48].

#### 3.1.2. Materials

The material composition of CUF is a critical factor that determines both its rheological properties during application and the final thermomechanical reliability of the assembly. Underfill is generally based on polymers. Typically, it is a mixture of a liquid organic binder and an inorganic filler. Epoxy resin is commonly used as an organic binder. Other binders used include cyanate esters or other types of resins. Microscopic silica particles are most often used as an inorganic filler. The purpose of using silica particles mixed into the epoxy binder is to improve the resulting properties of the material mixture both before and after its hardening. The number of fillers reaches up to 70% of the weight ratio. The mixtures may also contain other chemical elements such as dispensing agents, which ensure the uniform dispersion of inorganic filler particles within the polymer matrix and prevent them from settling or agglomeration; adhesion promoters; toughening agents; curing agents; and catalysts [5,6,18].

The quantity and type of fillers and chemical elements used affect a significant number of physical properties that affect the resulting properties of the underfill. The most important parameters include the modulus of elasticity, electrical conductivity, thermal conductivity, relative permittivity, strength, color toughness, and rheology. The most critical parameter in optimizing filler mixtures is the silica particle size. Particles that are too large will prevent capillary flow, and particles that are too small will increase viscosity and reduce the flow rate [17].

The compatibility and uniform dispersion of the fillers within the polymer matrix are two other critical interrelated factors. Proper compatibility ensures strong interfacial adhesion between each filler particle and the polymer matrix. This strong bond is essential for effectively transferring mechanical stress from the flexible polymer to the rigid fillers, which is the primary mechanism for reinforcing the composite’s mechanical properties. In parallel, uniform dispersion ensures a homogeneous distribution of these fillers. If the fillers are poorly dispersed, they may form agglomerations, leading to a non-uniform material with localized stress points that can compromise its reliability. These agglomerations also dramatically increase the underfill’s viscosity, hindering its flow and processability. Therefore, achieving high compatibility and dispersion through chemical surface modification of the fillers is essential for formulating a reliable underfill with low viscosity and predictable properties [49,50].

The resin matrix used for underfill is mostly epoxy-based. Cured or cross-linked epoxy forms thermosetting polymers, which usually have good thermal stability, chemical resistance, and mechanical properties. Typical resins for underfill binders are bisphenol A epoxy resin; bisphenol F epoxy resin, characterized by higher chemical resistance after curing; novolac epoxy resin, characterized by the formation of a highly cross-linked polymer network characterized by high temperatures and chemical resistance but low flexibility; aliphatic epoxy resin, characterized by low viscosity, which is often used to modify the properties of other epoxies; and glycidylamine epoxy resin, characterized by relatively low viscosity and good mechanical properties and heat resistance [6].

In their work, Ishibashi et al. [51] focused on the influence of silica fillers on the fatigue crack propagation rate of underfill resistance. The results show that resistance to fatigue crack propagation in underfill resin increased with increasing filler content. The work by Li et al. [52] focused on improving the material’s thermal conductivity; carbon fibers were used to replace silica particles, or a combination of both fillers was used. The results show that carbon fibers can increase thermal conductivity while achieving properties similar to those of storage modulus and CTE, as with silica particles. However, the electrically insulating properties rapidly deteriorate at a carbon content higher than 10%. Furthermore, Lee Sanches et al. [53,54] also investigated the possibilities of using hybrid Al_2_O_3_/BN and AlN/BN filler systems. Both techniques showed the option of using alternative fillers instead of classic silica particles, increasing the underfill’s thermal conductivity while maintaining good electrical insulating properties and low CTE [53,54].

A reduction in size of the SiO_2_ particles must be considered for gap-minimizing scenarios. With the rise in nanotechnology, silica nanoparticles began to be used in the underfill, which is essential to carry out appropriate surface treatments to prevent the formation of agglomerations, which would worsen the resulting properties. Lin et al. [55] dealt with the possibilities of surface treatment of the given particles for underfill materials. In contrast, other works, such as Peng et al. [56], investigated the effect of fillers, toughening agents, and coupling agents on the curing shrinkage of underfill. Volume epoxy shrinkage has an essential impact on the internal stress of the underfill, which is often associated with reliability problems such as deformation caused by the so-called “warpage” effect or cracks. The study by Peng et al. [56] found that adding SiO_2_ particles to epoxy reduces the overall shrinkage of the resulting material. Simultaneously, it was found that as the proportion of SiO_2_ particles increases, shrinkage during curing also decreases the material. However, a high density of silica nanoparticles can increase viscosity, which is an undesirable effect concerning the capillary flow of the underfill. Toughening agents significantly impact shrinkage, and most reduce the shrinkage effect due to steric hindrance. Coupling agents, except for the inert one, can also minimize shrinkage [56].

Among other essential elements contained in underfill materials are inorganic fillers, which, on the one hand, reduce coefficients of thermal expansion, reduce shrinkage during curing, improve thermal conductivity, or help to achieve specific mechanical properties. Currently, the most widely used are silica particles, which play an essential role in reducing the CTE and the cost of the resulting underfill material. Many types of silica exist, but fused silica is the most widely used underfill filler. Other essential components of underfill materials are catalysts, which serve as key elements in determining curing latency. The reason for using catalysts is that it is not desirable for the adhesive to cure at room temperature but for rapid curing to occur at the desired temperature. Other elements used are adhesion promoters, which are used to improve the adhesion between the cured underfill, the substrate, and the surface of the chip. Finally, underfill materials also contain toughening agents, the purpose of which is to improve the toughness and flexibility of cured underfill. Fillers generally modify and affect many key properties of the underfill material. These properties are rheology, the CTE, thermal and electrical conductivity, the dielectric constant, strength, toughness, the modulus, curing latency, and color [6,17].

#### 3.1.3. Benefits

Underfill improves the fatigue of solder joints in flip-chip assembly by redistributing stress caused by the CTE mismatch of the given materials to the substrate, solder joints, and chip. In addition, underfill helps to increase mechanical strength, resistance to moisture and external climatic influences, and reduce electromigration [5,17].

#### 3.1.4. Disadvantages

Despite its benefits, the conventional CUF method has several significant drawbacks that have led to the development of alternative technologies. The main problem is the slow process, which becomes a bottleneck in production, requiring several separate steps, such as cleaning, dispensing, and curing. This complexity also increases the risk of defects, especially incomplete gap filling and the formation of voids. These voids, which reduce the reliability of the assembly, have several leading causes: incompatibility with flux residues, evaporation of absorbed moisture, and imperfections in the dispensing and material flow process itself. Another critical factor affecting reliability is the deformation of the entire assembly (warpage), which occurs primarily during cooling after curing the underfill due to stresses caused by different CTEs between the materials [5,17,34,35,37,41,43,44,45].

### 3.2. No-Flow Underfill (NUF)

The principle of NUF, which is schematically compared to the CUF process in Figure 4, consists of modifying the chip connection process using flip-chip technology, which consists of several consecutive and interconnected steps, specifically the application of flux to the base, subsequent mounting of the chip, followed by reflow soldering, the cleaning of the assembly from the change, and then the process of applying the underfill and curing it [5].

In 1992, Pennisi and Papageorge (Motorola) patented connecting flux and underfill into one material [57]. This started the research and development of the no-flow process of underfilling [5]. In 1996, the first NUF process work focused to compare the standard chip connection process using the flip-chip technique was published. This process was characterized by depositing underfill on the base before placing the chip. After the subsequent installation of the chip, the entire assembly was soldered using reflow soldering, where solder joints are formed between the chip and the base, and simultaneous underfill curing. NUF, sometimes called fluxing underfill, is a modified approach that eliminates the separate application of flux and the cleaning process, subsequently applies the underfill; and cures it. Thanks to this improvement, leading to the merging of the process step of reflow soldering and curing the underfill, along with eliminating the time required to underfill the whole space between the chip and the substrate using capillary flow, it was possible to simplify the entire production process, increase its productivity, and reduce its costs [5,7,58].

The primary challenge for the successful implementation of NUF was the development of suitable materials, the first of which was patented by Wong et al. [59]. NUF’s two most critical properties are delaying the cure and acting as a flux material. Additionally, NUF should have several specific properties, including the following:

- A flux function to remove oxides on terminals and soldering surfaces, thus facilitating the creation of a high-quality solder joint.
- A delayed cure property, which allows the formation of a solder joint before the actual hardening of the underfill; gel underfill could prevent the melting of the solder bumps and their wetting and contact with the solder pads, preventing the formation of a solid mechanical and electrical connection.
- The possibility of curing within the standard production line during the reflow soldering process or offline at a temperature of less than 175 °C.
- The smallest possible filler particles, which are trapped between the formed solder connection.

Thus, mostly NUF materials are non-filled with filler particles [5,7].

#### 3.2.1. Process

**Figure 7:**
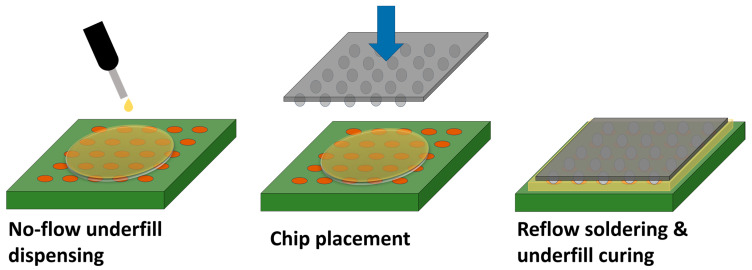
Schematic illustration of the process step of the no-flow underfill.

The manufacturing NUF process is schematically shown in Figure 7. There are three consecutive steps. Firstly, the required amount of underfill is applied to the base. Secondly, the chip is mounted on a substrate and pushed into the NUF; thus, its bumps and substrate solder pads come into contact. Finally, reflow soldering is performed. The underfill must first be cured if necessary to create solid solder joints between the chip and the substrate solder pads. Alternatively, the underfill can be cured after reflow soldering [5,7,27].

Modifying the NUF process made it possible to use filled materials, improving their properties and reducing the CTE. This modification became the so-called double-layer NUF, which consists of applying two different materials. The lower layer is made of materials without fillers and has higher viscosity. The upper layer is made of material containing fillers up to 65 wt.%. However, the use of conventional micron-sized fillers in a single-layer NUF is problematic, as they can be trapped between solder joints. Therefore, nanoparticles are required. The main challenge with nanoparticles is their dispersibility. Due to their large surface area, they tend to form agglomerations that significantly increase the material’s viscosity. Proper surface treatment is therefore critical to ensure a homogeneous, low-viscosity mixture that allows for a high filler content [5,60,61].

In the case of NUF, works such as those by Lee et al. [62,63] focused on the mechanism of creating voids in it. These works pointed out that the chemical reaction between the solder bumps and the underfill during the solder wetting and underfill curing process is one of the main factors for forming voids.

**Figure 8:**
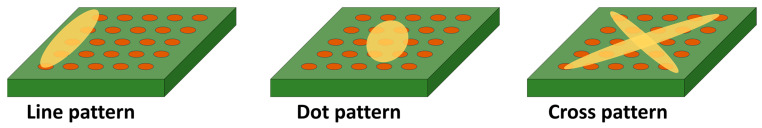
Schematic illustration of the investigated dispensing patterns by Colella et al. [64].

The optimization of the NUF process is investigated in the work by Colella et al. [64], who examined the influence of the NUF dispensing pattern. This work investigated three patterns: dots, lines along the edge, and crosses, as shown in Figure 8. This work found that the application pattern of NUF itself affects the formation and number of voids. The smallest number of voids was observed in the line pattern.

The works by Lee et al. [62,63] focused on investigating the causes of void formation in NUF in flip-chip assembly. Both articles indicated a chemical reaction between the solder and the underfill material during reflow. This reaction, accelerated by the heat from the endothermic reaction during solder melting, which leads to a concentration of heat in the vicinity of the solder, releases low-molecular-weight components such as fluxing agents and water, which become gaseous when heated, leading to the formation of voids [62]. Process parameters such as contact force, contact time, and substrate pretreatment have relatively little influence on the formation of voids, but the presence of solder is essential for their formation [63].

Material-based strategies have also been developed to mitigate void formation. Lee et al. [65] proposed using nano-sized fillers with low surface energy, such as Al_2_O_3_, within the NUF formulation. These nanoparticles act as numerous heterogeneous nucleation sites that distribute thermal energy during reflow, which prevents any single location from reaching the critical Gibbs free energy required for a macroscopic void to form and grow. Another approach, by Sung et al. [66], involved the application of a solvent-free “fluxing underfill”. This material minimizes outgassing by exhibiting significantly less weight loss during thermal processing, directly correlating to achieving a void-free interconnect.

#### 3.2.2. Materials

The composition of NUF material is similar to the composition of CUF. It generally consists of epoxy resins, hardeners, a curing accelerator, a self-fluxing agent, a viscosity-controlling agent, a coupling agent, and latent catalysts; however, it often contains little to no fillers, resulting in a higher CTE [5,59,67].

Similar materials to CUF can be used as epoxy resins, such as bisphenol A, bisphenol F, and novolac. Organic carboxylic acid anhydride is used as a hardener. Metal chelates, e.g., Co(II) acetylacetonate, are latent catalysts. The advantage of using metal chelates lies not only in their latent acceleration but also in their wide temperature range of curing. NUF contains only a small or no amount of inorganic fillers in the form of silica particles, which leads to a higher coefficient of thermal expansion and overall worse properties [5,27,59,67,68].

To optimize properties such as toughness and reliability, advanced resin systems are often developed by combining different epoxy types. Fan et al. [48] demonstrated a key strategy to improve toughness and reliability. Their work started with a base cycloaliphatic epoxy system, which, despite its processability, showed poor thermal shock performance and cracking, likely due to a brittle cured network. Blending this cycloaliphatic epoxy system with longer-chain solid epoxy resins significantly improved its reliability, with the new formulations surviving over 1000 thermal shock cycles. For other specific applications, such as high-temperature lead-free soldering, Tuan et al. [69] developed a specialized system based on a mixture of cycloaliphatic and bisphenol F epoxies, using organometallic salts as latent catalysts to prevent premature curing. A further significant advance is using cyanate ester resins, as described by Bao et al. [70]. These resins inherently offer a high *T*_g_, a high modulus, and a low CTE, but their use was historically limited by massive void formation until novel flux technologies enabled their successful application [69,70].

The main component of NUF is an integrated fluxing agent. While weak carboxylic acids or anhydrides have traditionally been used, their disadvantage is that they often act as plasticizers, reduce the resulting *T*_g_ of the cured material, and introduce risks associated with corrosion and moisture sensitivity. In response to these problems, Bao et al. [70] developed a new class of fluxing agents that actively react with the matrix resin, unlike traditional ones. This innovative approach eliminates void formation, especially in cyanate ester-based systems; accelerates curing; and significantly increases the resulting *T*_g_. Moreover, Rebibis et al. [71] confirmed in practice that the materials with the best results contained effective fluxing agents.

The key material challenge for NUF is balancing processability with thermomechanical performance, which is primarily dictated by the filler content. While traditional NUF materials contain little to no filler to avoid particle entrapment between solder joints, this results in a high CTE and reduced reliability [5]. Significant developments have focused on strategies to increase filler content. To achieve higher filler content while maintaining low viscosity, Tuan et al. [69] used a bimodal filler system by combining two different sizes of silica particles. A completely new function of fillers was introduced by Lee et al. [65], who proposed using nanoparticles to suppress void formation. These particles, such as low-surface-energy alumina, act as numerous nucleation sites that dissipate thermal energy and prevent the growth of macroscopic voids [65]. However, the success of this method is strongly dependent on the surface energy of the nanoparticles; high-surface-energy materials can, on the contrary, promote void formation [65]. Another material strategy to minimize void formation is an entirely solvent-free formulation, as demonstrated by Sung et al. [66], whose “fluxing underfill” showed minimal outgassing and weight loss during the remelting process.

#### 3.2.3. Benefits

The main advantage of NUF is the significant simplification and streamlining of the manufacturing process. This technology eliminates the separate steps of flux application, subsequent cleaning, and time-consuming capillary filling and curing, as it integrates all these functions into a single step along with solder reflow. This makes the entire process fully compatible with standard SMT production, increasing productivity and reducing manufacturing costs. In addition, modern NUF materials offer further improvements; for example, new types of fluxing agents that chemically react with the matrix resin can eliminate void formation, accelerate curing, and even increase the assembly’s resulting glass transition temperature [5,7,57,71,72].

#### 3.2.4. Disadvantages

Despite the process advantages, NUF technology presents specific disadvantages and challenges, mainly related to material composition and process risks. Since NUF materials often contain little or no inorganic fillers to prevent them from being trapped between solder joints, they typically have a higher CTE, which poses a potential risk to reliability. Another key challenge is the high probability of voids, which can be caused by outgassing, air entrapment, or chemical reactions between the solder and underfill during the reflow process. However, the most critical process risk is the need for precisely timed, so-called delayed polymerization. If the material gels before the solder has fully melted and the joints are formed, it could prevent proper wetting of the solder pads and lead to defective or missing joints [5,57,58,61,62].

### 3.3. Wafer-Level Underfill (WUF)

The next step, and an opportunity to improve the flip-chip manufacturing process, was to transfer the underfill to the wafer level. WUF, also called wafer-applied underfill, is an improved concept of NUF, where the underfill application takes place at the wafer level before dicing into individual chips. Many publications were devoted to this technology, as it was an opportunity to speed up and make flip-chip production cheaper while achieving high reliability. Developing this method and specific materials is possible based on a fundamental understanding of the underfill curing process. Furthermore, the possibility of underfill precoating at the wafer level made it possible to eliminate the underfill application within standard SMT production, thanks to which an SMT-compatible process was created [5,73,74].

WUF is another step that leads to increased productivity and reduced manufacturing process costs. It involves transferring the “encapsulation” using underfill to the silicon wafer level before slicing the wafer into individual silicon chips. From a process point of view, this is the most advanced but also the most demanding option, making it potentially the most effective and cost-effective. At the same time, this approach cancels the traditional division of the semiconductor industry into the “front end” and the “back end” as it is the first attempt to unite these two separate sectors [7].

Although transferring the underfill to the wafer level eliminates several process steps in current flip-chip manufacturing, the materials used must meet specific requirements to make the process feasible. The most important and essential requirement is that the WUF material on the wafer is non-tacky at room temperature to meet the criteria for dicing and storage. However, the need for a non-tacky solid encapsulant challenges attaching the chip to the substrate. Furthermore, overlapping solder bumps with filled WUF can lead to the entrapment of filler particles; unevenness on the WUF surface can lead to the formation of voids, and overall, there can be a risk of poor wetting to the substrate and a lack of filet formation. All these factors can lead to the deterioration of the final reliability [7].

#### 3.3.1. Process

Applying WUF is relatively simple and can be approached in two different ways. The first option involves applying the underfill after wafer bumping using techniques such as spin coating, screen printing, curtain coating, or meniscus coating. For these methods to produce a uniform coating, the material must have proper viscosity [75,76,77]. After coating the wafer with underfill, it must be partially cured (B-staged) to a state commonly known as the B-stage. Subsequently, the wafer can be diced into individual chips, which can be handled like standard chips and mounted on a substrate using standard SMT equipment. Due to the difficulty in chip alignment with applied WUF, most processes have bumps free of underfill resin or use a thin layer of resin that is not filled [76,77,78].

**Figure 9:**
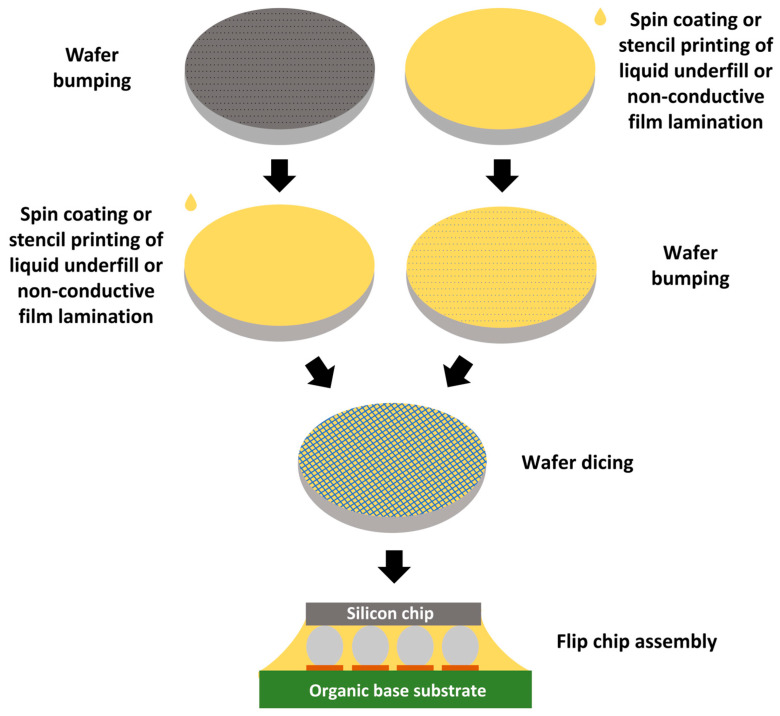
Process steps of wafer-level underfill (WUF) [5].

Another option to approach this first method is to apply an underfill to the wafer before the bumps are created. Subsequently, bumps are formed, and the underfill can serve as a protective mask. The following steps are the same as when underfill is applied to a bumped wafer. These two approaches for WUF application are schematically shown in Figure 9 [77].

Burress et al. [79] described the possibilities of applying WUF on the wafer before bumps were formed. First, the heavily filled underfill is applied to the wafer and then cured. After curing, a laser is ablated to form microvias that expose the wafer bond pads. Subsequently, these vias are filled with solder and bumped.

With the development of nanocomposites, a concept of photo-definable material was introduced by Sun et al. [80], which can act as both a photoresist and WUF. These materials are photosensitive polymer composites that function as a negative tone photoresist. To maintain optical transparency, 20 nm silica particles were used as the filler. They contain a photo-initiator that releases cations after UV exposure through a mask, which in turn initiates the crosslinking reaction of the epoxy. Because crosslinking makes the exposed areas insoluble, a subsequent development step removes the unexposed material, leaving a patterned underfill layer with openings for solder bumping. Unfortunately, post-thermal curing was still necessary for full crosslinking of the epoxy. This technique brings another possibility of applying underfill to an unbumped wafer [80].

One of the most common methods is applying a liquid material, often using spin-coating or stencil printing technologies. With this method, it is crucial to properly set the process parameters based on the material’s viscosity and rheological properties to ensure a uniform layer of thickness and clean scribe lines for subsequent wafer dicing [81,82].

An innovative option, introduced in 2002 by Chaudhuri et al. [83], is the application of underfill as a thin, dry film, referred to as a non-conductive film (NCF). The principle consists of laminating a thin film of a polymer mixture heavily filled with silica particles onto a wafer. After lamination, the tops of the bumps must be exposed, for example, using chemical mechanical planarization. The main advantage of this method is the elimination of the complex B-staging process and the minimization of potential particle segregation. Lamination is performed in a vacuum to prevent the formation of voids and defects [81,83].

The WUF process developed by IBM is called over-bump applied resin (OBAR). In this process, the filled resin is applied over the bumps of a wafer. The resin is subsequently B-staged so that the wafer can be diced into separate chips. The resin flows outward during chip bonding, allowing contact and joining between solder bumps and substrate pads. This filled resin that flows outwards creates a filet around the chip [84,85].

The coated volume of underfill must be selected appropriately, as a thick layer of coated underfill may provide sufficient material to ensure good fileting. Still, on the other hand, it may increase the buoyancy forces during reflow, lifting the chip away from pads, and poor joint formation or non-contact will result in such cases [76].

As the pitch of the solder bumps becomes finer and the standoff height becomes smaller, the mass reflow technique used to create the solder joints between the flip chip and the substrate becomes problematic. A viable method of connecting flip chips with ultra-fine pitch within advanced encapsulation is the thermo-compression (TC) bonding technique. This bonding technique can be further divided according to the processes it follows. These processes involve a non-conductive paste (NCP), underfill NCF, and dip fluxing without pre-applied underfill, all followed by TC bonding [86,87].

#### 3.3.2. Materials

Specific requirements are placed on WUF materials. On the one hand, the materials must be able to cure during reflow and should not gel before the solder melts. At the same time, it must be sufficiently solid and possess sufficient mechanical integrity and stability after the B-stage so that the wafer with WUF can be diced without damaging the WUF and withstand handling and storage [77,88].

WUF can be divided into two types, both from the process’s and materials’ points of view. The first type enters the process in its liquid form and is often applied to the wafer using spin coating. The second type enters the process as a film and is laminated onto the wafer [81]. A filler, most often silica, is the key component for modifying thermomechanical properties. Its addition reduces the CTE and increases the elastic modulus. As reported by Katsurayama et al. [89], the filler content can reach up to 60% by weight. An innovation of recent years was presented by Prabhakumar et al. [90] and Sun et al. [80]: the transition from micron-sized fillers to nanoparticles (20–150 nm in size). Nanofillers improve the material’s optical transparency for machine vision systems, reduce the risk of particle trapping in fine joints, and enable the development of photo-definable underfills.

#### 3.3.3. Benefits

The main advantage of WUF technology is the significant simplification and streamlining of the manufacturing process, which leads to increased productivity and reduced overall costs. Applying underfill in a batch at the wafer level eliminates the time-consuming steps associated with individual chip treatment, such as capillary filling, flux cleaning, and separate curing. In addition, the method is fully compatible with the standard SMT process, which significantly facilitates its integration into existing production lines [5,7,74,75].

In addition to the advantages of the process, WUF also provides key technical protection for the chip from mechanical stress during the soldering process itself. As Feger et al. [85] describe, this prevents, for example, the formation of defects in the dielectric layer, which are known as “white bumps.” These properties make WUF technology more scalable for modern large-area, fine-pitch packages, where traditional CUF and its flow become slow and unreliable [5,84,85,91].

#### 3.3.4. Disadvantages

Despite its advantages, WUF technology has several significant drawbacks that complicate its implementation. The primary limitation is the demanding and often conflicting requirements for material properties. After application and partial curing (B-stage), the material must be strong, non-sticky, and mechanically resistant for subsequent wafer separation. However, it must become liquid again with low viscosity during final soldering to properly form solder joints [5,92].

There are several significant risks associated with this process. As detailed by Nah et al. [84], there is a high probability of voids, which can be caused by solvent outgassing, moisture release from the substrate, or air entrapment during chip assembly. Another complication is chip alignment, as the applied underfill often overlaps the solder balls, making them difficult for machine vision systems to detect. In addition, filled materials are at risk of filler entrapment between the solder ball and the contact pad, which can lead to unreliable connections. The entire process is generally difficult to control and has a very narrow process window, especially regarding B-stage control and soldering parameters. Also, incorrect material rheology can lead to insufficient filet formation around the chip, negatively affecting its final mechanical strength and reliability [5,7].

### 3.4. Molded Underfill (MUF)

**Figure 10:**
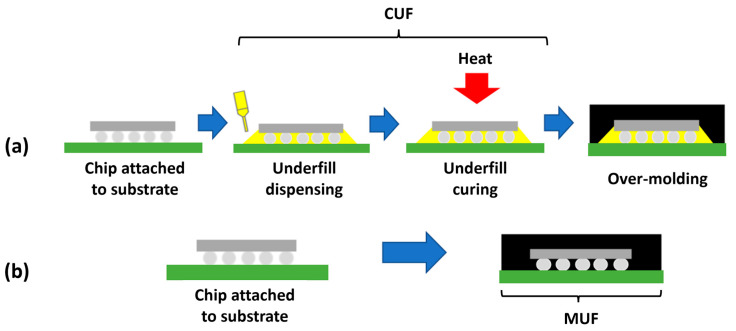
A schematic comparison of the (**a**) CUF and (**b**) MUF process steps [5,93].

MUF represents another significant technological advancement in flip-chip packaging, responding to the growing market demand for higher productivity and lower manufacturing costs, unlike the traditional, sequential two-step process that involves first applying CUF, then curing it, and then separately molding the chip (over-molding). MUF integrates these two steps into a single process, as schematically shown in Figure 10, in comparison with the CUF process. During this process, the molten epoxy molding compound (EMC) is injected under pressure into the mold, where it simultaneously fills the narrow gap between the chip and the substrate and forms a protective housing around the chip [93,94].

This pressure-controlled filling mechanism overcomes the physical limitation of slow capillary flow, a significant bottleneck in the conventional CUF process, especially for large chips or packages with a small gap (standoff). MUF technology has become an attractive alternative for a wide range of applications, from mass-produced packages for mobile devices to complex heterogeneous structures such as fan-out wafer/panel-level packages and advanced 2.5D/3D packages. Although this approach brings significant process and economic advantages, it also places new, specific demands on process control, material properties, and package design, with the most critical challenges of warpage control and void elimination remaining [95,96,97].

#### 3.4.1. Process

The basis of MUF technology is the transfer or compression molding process, which is adapted to fill micrometer gaps under the chip. To prevent the formation of voids, vacuum molding systems became a standard. These systems suck air from the mold cavity before injecting the material, thereby dramatically reducing the risk of its subsequent trapping in the melt. As stated by Joshi et al. [93], vacuum-assisted molding allows reliably filling even for tiny gaps of the order of 50 µM without defects [97,98,99,100,101].

Guo et al. [102] discussed the vacuum effect in detail, investigating its effect using a specially designed transparent mold that allowed direct flow visualization. They found an optimal vacuum level for each type of material. Too high a vacuum can cause an undesirable fluid front rupture in some materials, paradoxically leading to new, large cavities. The use of a vacuum is, therefore, a key but, at the same time, sensitive part of the process, the optimization of which is necessary to achieve the high quality and reliability of the package.

A key phenomenon in the MUF process is the uneven material flow. The material flows significantly faster in the wider channels around the chip than in the narrow, resistive gap below it. This leads to the flow fronts connecting behind the chip before filling the space below it, trapping an air pocket. Lee et al. [103] confirmed in their simulation study that the pressure inside the resulting cavity gradually equalizes with the pressure of the surrounding melt. This explains why the cavities near the vent channels, where the melt pressure is lower, are usually larger. Precise adjustment of the process parameters is essential to control this complex flow. Optimizing the molding pressure is a trade-off: a high pressure helps eliminate cavities but can damage sensitive structures on the chip or cause solder joints to stretch. Similarly, mold temperature affects viscosity and cure speed—too high a temperature can lead to premature solidification of the material and under-pouring of the mold [100,101,103,104,105,106].

In response to these challenges, advanced process techniques have been developed. Chee et al. [79] and LeBonheur et al. [87] described using a highly compressible film inserted between the die and the mold for exposed-die packages. This film protects the die surface from mold flash and compensates for minor differences in die height and tilt, preventing damage during molding. For wafer-level applications, Mok et al. [99] demonstrated that optimizing the pattern of material deposition on the wafer before molding is key to achieving void-free filling. The mold design itself is also critical. Lee et al. [105] found that even different gate types and locations may not be sufficient to eliminate voids and, therefore, proposed an optimized mold vent design to remove them effectively.

#### 3.4.2. Materials

The materials for MUF are highly specialized epoxy molding compounds, the composition of which is a compromise between the conflicting requirements for flowability and final thermomechanical properties. The primary requirement is the ability of the material to fill the narrow gaps under the chip, which requires low viscosity and, above all, a small filler particle size. The maximum size of the filler particles should not exceed one-third of the height of the gap under the chip to avoid trapping and blocking the flow [93,94,107].

For the reliability of the package, the cured material must have a low CTE, which is as close as possible to the CTE of the substrate (15–25 ppm/°C). This minimizes stress and warpage arising from the differential expansion of the silicon chip and the base substrate. A low CTE is achieved by a high inorganic, often silica, filler content, which can constitute more than 80% of the material weight. However, the high filler content dramatically increases the viscosity of the melt. Material manufacturers solve this key problem by using fillers with an average particle size in the order of micrometers and optimizing their size distribution. For example, in their study, Kamimura et al. [89] showed that by optimizing the particle size distribution, viscosity can be reduced by up to 70% while maintaining a high filler content of 83.5 wt.% [93,94,95].

Understanding the material’s viscoelastic properties is crucial for accurately predicting the behavior of the bushing, especially its deflection. As described in detail by Lin and Lee [108] and Yeh et al. [109,110], the relaxation behavior of the MUF material during and after curing significantly affects the final deformation of the bushing, and it is, therefore, essential to include it in simulation models.

#### 3.4.3. Benefits

The main benefits of MUF technology are significant production efficiency, cost reduction, and greater flexibility in package design. Combining the underfill and final encapsulation processes into a single step eliminates the time-consuming operations associated with capillary underfill, such as dispensing, underfill flow, and separate curing. This, together with the ability to batch process entire strips or wafers, leads to a radical increase in throughput and a reduction in manufacturing costs [93,98,105].

From a design perspective, MUF allows for denser component integration because it does not require a vast filet space around the chip. Joshi et al. [93] report that the required chip spacing can be reduced from 0.8–1.0 mm for CUF to just 0.3–0.4 mm for MUF. This allows for a smaller overall package size, saving expensive substrate materials. Finally, MUF can also contribute to higher reliability. The highly filled materials used have a lower CTE, leading to better control of package deflection and stronger mechanical protection for solder joints [95,111].

#### 3.4.4. Disadvantages

Despite its benefits, MUF technology faces several significant challenges. The risk of under-chip voids is the most important and frequently investigated issue. These defects are primarily caused by non-uniform material flow, where the melt flows around the chip faster than under it, leading to air entrapment. A second critical issue is warpage, caused by volume shrinkage of the material during curing and CTE mismatch between individual components, which negatively affects downstream processes such as mounting the encapsulated component onto a PCB during SMT [99,101,105,107,108].

Other specific issues include material and process defects. Materials with fine fillers and low viscosity, which are necessary for good flow, are prone to mold flash. With highly filled materials, filler segregation can occur during flow, leading to inhomogeneous properties across the package. Poor process control can lead to physical defects such as blisters or pits on the substrate. Even if the package is successfully manufactured, reliability failures such as corner cracks or delamination at the material interface can occur in stress tests. According to Joshi et al. [93], MUF technology also has limitations. It is not ideal for applications with huge chips or excellent solder joint pitches, where capillary underfill still offers more reliable results [93,94,98,112].

### 3.5. Board-Level Underfill/Second-Level Underfill

**Figure 11:**
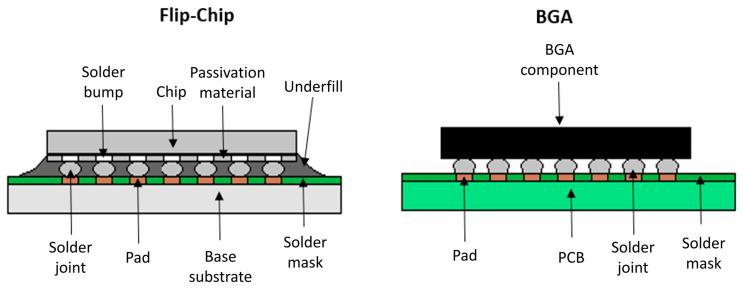
Comparison of a flip-chip assembly and a BGA assembly. Both technologies use a topologically similar principle: solder bumps on the bottom side of the component (a bare chip or package) connect it to an organic substrate [5,20].

As the miniaturization trend continues and the input and output density of ball grid arrays (BGAs) and chip-scale packages (CSPs) increases, their physical parameters, such as pitch and stand-off height, are increasingly approaching those of the original flip-chip technologies. This convergence is further highlighted by their fundamentally similar assembly structure, where a component is attached to an organic substrate via an array of solder joints on its bottom side, as shown in Figure 11. As a result, these components face similar reliability challenges, especially concerning thermomechanical stresses caused by the different coefficients of thermal expansion (CTEs) between the package and the PCB. As specified in the IPC J-STD-030A standard, board-level underfill is used to alleviate these stresses and increase the mechanical strength of the assembly. Reliability becomes critical, especially in harsh operating environments such as the automotive and aerospace industries or portable devices exposed to mechanical shock, vibration, and bending. It has been shown that while CSPs often pass thermal cycling without additional protection, they fail drop tests, making underfill necessary to ensure their mechanical integrity [19,113,114,115,116].

Although the primary goal of applying underfill is to improve mechanical resistance, its presence creates a complex interaction between materials that can also have adverse effects. It has been shown that while underfill significantly increases drop and vibration resistance, it can paradoxically reduce reliability during thermal cycling, especially if the material used has a CTE substantially different from that of the soldered joints, which is usually the case with underfill materials with a low filler content. This trade-off has led to the development of alternative strategies, such as partial CUF (PCUF) or edge/corner bonding, which try to find the optimal balance between reliability, cost, and process time [113,114,117,118,119].

#### 3.5.1. Process

The basic process method for applying underfill at the board level remains CUF, where a low-viscosity polymer material is applied along one or more edges of the mounted component and is drawn into the gap between the package and the board by capillary action. The boards are often preheated to ensure optimal and rapid flow. The IPC J-STD-030A standard [20] describes various dispensing patterns, from the classic “I” or “L” shape to the “U” shape, cautioning that faster patterns such as the “U” shape have a higher risk of air entrapment and void formation. Stippling or smaller “L” patterns are used at the package’s corners [100,103,104,105] for partial application.

A key challenge in the process is quality control. One of the most significant problems is the formation of voids, which can be caused by several factors. As the IPC standard emphasizes, it is essential to thoroughly dry the board before applying underfill, as absorbed moisture evaporates during curing and causes voids [20]. Plachý et al. [120] have shown that even exposing the board to an environment with a relative humidity of 30% leads to a significant increase in voids. Another cause is flux residues, which can physically block the flow or chemically interfere with the curing process [115,121]. However, the detection of these voids is not a simple task. Visual inspection is limited to the outer filet to diagnose these defects. X-ray tomography has proven unsuitable due to low contrast and solder artifacts [122,123]. However, the flip-chip standard, scanning acoustic microscopy (SAM), is almost ineffective for BGAs at the board level because the component substrate strongly attenuates the signal. Perraud et al. [123] proposed an advanced destructive method combining planar grinding with subsequent SAM analysis.

Considering the dispensing processes’ complexity and time-consuming nature, alternative procedures have been developed at the board level, such as using NUF. Another important aspect is rework ability, which is key at the board level. As described in IPC J-STD-030A, the repair process involves local heating, mechanical removal of the component, and subsequent cleaning of underfill residues, which is a demanding process with the risk of damaging the board. However, reworkability is usually only possible for underfill materials with a low filler content [20,124,125].

#### 3.5.2. Materials

The material composition of underfills for BGA and CSP applications is crucial for their final function and reliability. They are typically based on two-component, pre-mixed, and frozen epoxy systems. The main component is usually epoxy resins, which are filled with inorganic fillers, most often silica. Overall, the composition is similar to the CUF materials used for flip chips. The filler’s main task is to reduce the resulting material’s CTE to make it as close as possible to the solder’s CTE, typically around 25 ppm/°C, and the substrate, thereby minimizing thermomechanical stress. The key parameter is the size of the filler particles, and it is recommended that the maximum diameter of the particles should not exceed one-third of the size of the smallest gap to prevent them from jamming and filtering during flow [5,20,21,114,126].

The suitability of an underfill for a particular application is determined by its key thermomechanical properties: the CTE, *T*_g_, and Young’s modulus. A low CTE is essential for high reliability in thermal cycling, as high thermal expansion of the material can reduce the life of solder joints due to the induction of additional stress. A high *T*_g_ is also key, which must be above the operating temperature, as exceeding it softens the material and leads to failure. The effect of the Young’s modulus on the technology is complex. While a higher modulus is advantageous for thermal cycling resistance, a lower modulus is better at absorbing energy and is preferred for resistance to mechanical shock and drops [20,114,117,119,126,127,128,129,130].

Special underfills are being developed for applications requiring repairability. These materials are designed to soften or degrade the chemical structure when heated to the repair temperature, significantly decreasing bond strength and allowing for easy component removal. However, the price of repairability often has poorer thermomechanical properties, such as a lower *T*_g_ and a higher CTE, due to the lower filler content [20,114,119,125,127,131].

#### 3.5.3. Benefits

The primary and most frequently documented benefit of applying underfill to BGAs and CSPs is a dramatic increase in the mechanical durability and reliability of the assemblies. The most significant improvement is observed in drop and shock resistance, where studies consistently demonstrate a multi-fold increase in the life of solder joints. For example, Liu et al. [115] reported a 5- to 6-fold improvement in reliability in a drop test. Similarly, significant improvements in resistance to vibration and repeated bending have been demonstrated, which are key parameters for portable devices and automotive electronics. Underfill acts as a shock absorber and stiffener that locally reinforces the assembly, reduces board bending, and effectively redistributes stress from individual solder joints to the entire package surface [19,20,116,126,131].

#### 3.5.4. Disadvantages

Despite its undeniable benefits, applying underfill at the board level brings several significant disadvantages and technological challenges. The most crucial trade-off is the reduction in reliability during thermal cycling that some underfill can cause. High-CTE materials induce additional axial stress in solder joints, which accelerates material fatigue and paradoxically shortens the life of the assembly. Another critical disadvantage is the increased mechanical stress on the chip itself. Underfill creates a rigid mechanical connection that transfers energy directly to the silicon chip upon impact, which increases the risk of its breakage, especially in modern thin packages [114,117,131,132].

From a process perspective, the main disadvantages are complexity, time, and cost, as dispensing and curing are additional steps that require expensive specialized equipment and can become a bottleneck in production. Inextricably linked to the process is the formation of voids, a serious defect most often caused by moisture in the board, flux residues, or imperfections in the material flow related to the dispensing process. The last major challenge is repairability issues. Standard epoxy underfills are non-removable after curing, which makes it impossible to replace a defective component and can lead to the scrapping of the entire, often expensive, board. Materials intended for easy reworkability are designed to soften significantly when heated, which is usually associated with a lower *T*_g_ and a higher CTE [115,117,120,121,124].

### 3.6. Alternative Localized Reinforcement Methods: Edge Bonding, Corner Bonding, and Partial Underfill

With increasing demands on the reliability of electronic assemblies, especially in portable devices, alternative local reinforcement methods have been developed in addition to traditional area underfill. These approaches, including edge bonding, corner bonding, and partial underfill, represent targeted solutions for increasing the mechanical resistance of BGAs and CSPs. The primary motivation for their introduction was the desire to reduce costs and shorten the production time, as underfill is often process-intensive and, for many applications, provides a higher level of protection than necessary. These methods are particularly relevant for consumer electronics, emphasizing manufacturing efficiency and repairability [20,119,133,134,135,136,137,138].

Although edge and corner bonding are sometimes used interchangeably, they have subtle differences. Corner bonding typically refers to applying adhesives in the form of drops or small patterns directly into the package’s corners. Edge bonding normally involves applying adhesives along a portion of the edge, often in an “L” shape, reinforcing both the corner and the adjacent edge. A specific variant is PCUF, where the CUF intentionally flows into the corners only, surrounding several rows of solder balls at each corner. These techniques represent a compromise between mechanical reinforcement and the impact on the thermomechanical reliability of the assembly, which is strongly dependent on the material’s properties [20,119,133,135,136,137,139].

#### 3.6.1. Process

**Figure 12:**
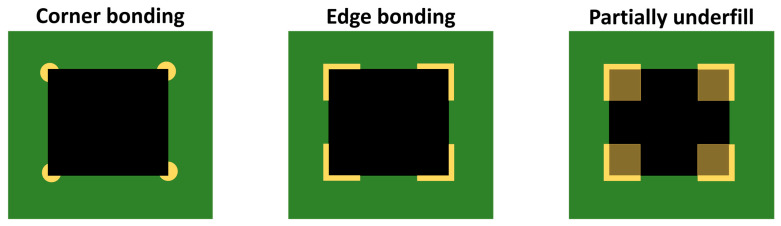
A schematic representation of corner bonding, edge bonding, and partially underfill.

The advantage of these alternative methods is their simplicity and flexibility over underfill. The adhesive is applied locally only to the perimeter of the package using a dispenser. Common application patterns are single drops in the corners (corner bonding) or an “L” shape covering the corners and parts of the edges (edge bonding), as shown in Figure 12. The IPC J-STD-030A standard warns against using single dots due to the risk of high-stress concentrations. The timing of the process varies: the adhesive, typically a corner bond, can be applied before the component is mounted and cured together with the solder during reflow or, more typically, for an edge bond only after the soldering process with subsequent separate curing by heat or UV radiation. Adhesives for these applications are designed to limit capillary flow and prevent the complete filling of the space under the package, except for the so-called “corner fill” or “partially underfill”, where partial leakage into the corners or beneath the component is desirable [117,119,133,135,136,137,138,139].

The key advantage is significantly easier reworkability. Since significantly less material is used, mechanical removal of the defective component is faster, with less risk of damage to the printed circuit board. From a process perspective, it is also advantageous that it is not necessary to pre-bake the PCB before edge or corner bond applications, which is usually required with underfills to prevent the formation of voids [22,117,119,133].

#### 3.6.2. Materials

The material composition of edge and corner bonding adhesives is crucial to their ultimate performance. These are often epoxy or acrylic systems, which can be unfilled or filled with inorganic particles such as silica. Adding fillers generally reduces the CTE and increases the material’s modulus of elasticity. Unfilled materials, which are often used for mobile applications, have a higher CTE, which is disadvantageous for thermal cycling, but their lower modulus of elasticity better absorbs mechanical shocks, drops, and vibrations [119,128,139,140]

#### 3.6.3. Benefits

The primary benefit is a dramatic increase in the mechanical strength of the assembly. Studies consistently demonstrate multiple-fold increases in the life of solder joints in drop and mechanical shock tests. Farris et al. [140,141] reported a 5- to 8-fold improvement in reliability in drop tests for edge-bonded components. Tian et al. [119] documented a 3- to 4-fold improvement for corner bonding. The adhesive acts as a mechanical bridge that locally stiffens the assembly, reduces board deflection, and effectively redistributes stress from critical corner joints over a larger area [22,117].

Another significant advantage is the already mentioned lower cost, faster manufacturing process than underfill, and easier repairability of defective components. Although this is not their primary purpose, these methods can also improve reliability during thermal cycling under certain conditions. This can only be achieved by carefully selecting a material with a low CTE and a high *T*_g_ [131]. For example, Shi et al. [139] found that a low-CTE corner bond extended the life by 1.3 times compared to the unbonded version.

#### 3.6.4. Disadvantages

The most significant risk and main trade-off when using these methods is the possible reduction in reliability during thermal cycling. High-CTE materials, which are common for these applications, induce additional axial stress into the soldered joints during temperature changes, which can accelerate material fatigue and paradoxically reduce the life of the assembly below that of the unreinforced variant [117]. Lee et al. [131] and Shi et al. [136,139] demonstrated that inappropriately selected adhesives, e.g., high-CTE acrylate, led to significantly earlier failure than specimens without it.

Another disadvantage is the change in stress distribution and the shift in the critical failure point. Reinforcing the corners can cause maximum stress to move directly from the area under the die to the corner solder joints. Since there are fewer joints in the corner to distribute the load, this can lead to their accelerated failure. Furthermore, the protection and reliability enhancement level is less comprehensive than an underfill. These methods do not protect the entire area under the package from environmental influences such as moisture or chemicals, and the overall mechanical enhancement is usually lower than with fully underfilled packages. The study by Akbari et al. [22] also showed that the path of crack propagation in the PCB itself can change depending on the curing temperature and the resulting residual stress [20,117,139,142].

## 4. Discussion

Underfill encapsulation methods have developed significantly, as shown in the previous chapter and summarized in Table 2 and Table 3. This development was not accidental but was a direct response to two key and often conflicting requirements of the electronics industry: maximum reliability and maximum manufacturing efficiency. The discussion of individual technologies can thus be framed as a search for the optimal balance between these two poles for different types of applications.

The original problem that underfill was designed to solve was purely mechanical, compensating for stresses arising from the different CTEs between a silicon chip and a cheaper organic substrate. CUF was a straightforward and highly effective answer. The principle of filling the space under the chip with a highly filled, low-CTE epoxy is the gold standard regarding reliability. Redistributing stress from fragile solder joints across the chip surface dramatically increases fatigue life.

Therefore, from an application perspective, CUF is still the method of choice where reliability is critical and cannot be sacrificed. This applies, for example, to large and expensive chips in servers, telecommunications infrastructure, or other high-end devices where failure in the field would have fatal consequences. However, the price for this reliability is, as shown in Table 2, a slow and expensive process that requires separate flux cleaning, dispensing, and curing steps. This bottleneck process has become the main driver for developing subsequent generations of underfill.

Technologies such as NUF, WUF, and MUF represent a fundamental shift in thinking. Instead of an additional step, they seek to integrate encapsulation into existing processes. The common goal of this method is to accelerate production and reduce costs radically.

NUF accomplishes this integration at the SMT line level, combining the functions of flux, underfill, and curing with the solder. This makes it an ideal candidate for mass, low-cost production where speed is key. However, this price is a significant trade-off in material properties as NUF materials are often unfilled, leading to a high CTE and potentially lower thermomechanical reliability. WUF takes integration to the next level, to the wafer level itself. It eliminates the underfill process from the final assembly, making it the most cost-effective approach for the mass production of standardized fine-pitch packages. MUF offers an elegant solution for more complex structures such as system-in-package and other 2.5D/3D packages, where it fills the space under the chips and creates the final package in one step.

However, these pre-applied methods shift the challenges from flow dynamics, the problem associated with CUF, to materials engineering and process control. The precise timing of cure for NUF and WUF, or control of melt flow to avoid voids for MUF, becomes critical. As summarized in Table 3, the risk of manufacturing defects is often higher, and repair is often impossible.

As BGA and CSPs became the standard and their dimensions became smaller and closer to the flip-chip dimensions, the same reliability problem emerged at a new level: at the junction between the package and the final printed circuit board. However, here again, application requirements differ.

Board-level underfill is essentially an application of the CUF principle to BGA/CSPs. Its goal is to ensure maximum resistance, not only thermomechanical but especially against external influences such as drops, vibrations, and bending. It becomes essential in industries with harsh operating conditions, such as the automotive and aerospace industries.

Edge and corner bonding represent a pragmatic compromise. For many consumers and mobile electronics, absolute hermetic protection is unnecessary, but a specific increase in drop resistance is needed. These localized methods provide just that—at a fraction of the cost and time of underfill. Their main advantage is the targeted reinforcement of critical corner joints. However, this approach does not protect against the external environment and can degrade the lifespan during thermal cycling if the material is poorly selected.

This analysis reveals that no single underfill method is universally superior; the selection is invariably a trade-off dictated by the specific application requirements. A designer must determine the primary driver for their product: whether it is absolute reliability, which favors CUF; manufacturing speed and cost, addressed by NUF and WUF; a high degree of integration, where MUF excels; targeted mechanical reinforcement for BGA and CSPs at the board level for use in harsh environments (board-level underfill); or simply a focused reliability improvement against mechanical shock, such as drops and vibrations (edge/corner bonding).

Future developments will likely follow the trends outlined in the text: the development of advanced materials with nanofillers to fill ever smaller gaps, process improvements to minimize defects in integrated methods, and deeper integration of MUFs into complex 3D structures, which represent the next step in the miniaturization of electronics.

## 5. Conclusions

Our comprehensive review has systematically analyzed and compared several key underfill encapsulation technologies in microelectronics manufacturing, as summarized in Table 2 and Table 3, concluding that their evolution and application are governed by a fundamental trade-off between their thermomechanical performance of the final polymer composite and its processability during manufacturing. The novelty of this work lies not merely in describing individual methods but in establishing a clear decision-making framework that connects strategic manufacturing goals with their underlying materials engineering challenges.

The key finding is that the choice of technology represents a selection between two primary strategies. The first, represented by capillary and board-level underfill, prioritizes maximum reliability, as highlighted in Table 3. This is achieved using highly filled epoxy composites with a low CTE to minimize thermomechanical stress. However, the cost of this approach is a slow process limited by the material’s rheological properties, especially its viscosity-dependent capillary flow.

The second strategy, encompassing integrated, pre-applied methods such as NUF, WUF, and MUF, prioritizes maximum manufacturing efficiency and low costs. This approach, however, imposes entirely different and often conflicting demands on material science. NUF requires limiting the filler content, which leads to a higher CTE and potentially lower reliability. WUF demands complex resin chemistry control to achieve a stable B-stage. MUF depends on optimizing melt rheology and the filler particle size distribution to prevent defects like under-chip voids.

Alternative methods like edge and corner bonding have emerged as a third, pragmatic path. These represent a targeted, low-cost solution for a specific failure mode: mechanical shock. The material selection is tailored to absorb energy, sacrificing comprehensive thermomechanical protection. The critical contribution of this analysis, therefore, is to frame the selection of an underfill technology as a complex, application-driven decision that must balance the demands for final part/assembly reliability governed by the cured composite’s properties with the capabilities and risks of the manufacturing process governed by the polymer’s liquid-state properties.

Future developments in underfill technologies will likely proceed along several key and interconnected paths. In the materials domain, research will focus on advanced nanocomposites capable of filling ever-smaller gaps in 3D packages and multifunctional fillers designed to simultaneously reduce the CTE while increasing thermal conductivity for more efficient heat dissipation. Furthermore, driven by sustainability trends, research will increasingly focus on eco-friendly alternatives like bio-based or easily reworkable resin systems to minimize electronic waste. In parallel, process optimization will see a growing importance of advanced modeling and simulation. These models will extend beyond predicting defects like voids and warpage to enable detailed simulation of curing kinetics. This will allow for adaptive curing profiles tailored to specific components, thereby minimizing thermal stress and extending their operational lifespan. Finally, this effort will be supported by the development of advanced diagnostics for both real-time, in situ quality monitoring and the reliable identification of material chemical compatibility, a crucial factor in defect prevention.

## Figures and Tables

**Table 1:** Selected thermomechanical properties of selected materials in electronic packaging [8,9,10,23,24,25].

Material Category	Material	CTE (ppm/°C)	Thermal Conductivity (W/m·K)	Young’s Modulus (GPa)
Semiconductors	Silicon	2.5–2.8	150	107–161
	Silicon carbide	3.8	120	450
	Gallium arsenide	5.7	46	86
Metals and Alloys	Copper	16.5–16.8	380–390	124
	Aluminum	23.5–23.8	210–235	69
	Gold	13.8–14.2	293	82
	Silver	19.0–19.7	418	76
	Nickel	14	92	221
	Solder (SAC305)	~22.0	~54	49
	Solder (Sn63/Pb37)	23.0–25.0	50	14
	Kovar (FeNiCo)	5.5	17.1	138
Ceramics	Alumina	6.4–8.8	22–30	300–390
	Aluminum nitride	2.9–4.6	195–319	345
Polymers and Composites	Epoxy resin (unfilled)	26.0–80.0	0.3	2.6
	FR4 (X-Y plane)	15.8–24.0	0.29	27.9
	FR4 (*Z*-axis)	80.0–90.0		12.2
	Polyimide (unfilled)	40.0–50.0	0.2	3.1–3.5
	Molding compound	18.0–65.0	0.67	8.9–28.5

**Table 2:** Comparison of key properties of underfill materials across different methods.

Property	CUF	NUF	WUF	MUF	Board-Level	Edge/Corner Bonding
Classification	Post-applied	Pre-applied	Pre-applied	Pre-applied	Post-applied	Post-applied
Typical application	Flip chip (post-assemble)	Flip chip (pre-assembly)	Flip chip (wafer-level)	Flip chip (molding process)	BGA and CSP (post-assembly)	BGA and CSP (localized)
Typical material base	Epoxy resin	Epoxy resin	Epoxy resin	Epoxy molding compound (EMC)	Epoxy resin	Epoxy or acrylic resin
Filler content	Up to 70 wt.%	Little or none <50 wt.% nanoparticles <65 wt.% double layer	Up to 60 wt.% micro to nano	Over 80 wt.% (fine filler)	Variable, high comparable to CUF, and lower for reworkability	Variable, often unfilled or with low filler content
Curing temperature	Up to 160 °C	During reflow	B-stage + during reflow	During molding process	Similar to CUF (must be adapted to the assembly)	Thermal (lower than CUF) or UV
Curing time	Slow (time-consuming)	Fast (during reflow)	B-stage (partial) + reflow	Fast (during molding cycle)	Slow (snap curable materials—a few minutes)	UV fast Thermal slow
*T*_g_	High (after curing)	Lower (unfilled), reduced by plasticizers	High (after full curing)	High	High (above operating temp.)	Can be lower (for reworkability)
Modulus	High	Lower (unfilled)	High	High	Complex (higher for TC and lower for drop resistance)	Lower (better for shock absorption)
CTE	Low, close to the solder (~25 ppm/°C)	High (due to low amount or lack fillers)	Low (high filler content)	Very low (highest filler content)	Variable and higher for reworkable types	Typically high
Key process control parameter	Viscosity and surface tension (capillary flow)	Latent catalysis (delayed cure)	Properties after B-stage (non-tacky and stability)	Melt viscosity and mold flow	Viscosity and wettability	Thixotropy (to limit flow)

**Table 3:** Application-oriented comparison of underfill technologies.

Method	Key Selection Drive	Typical Applications	Advantages	Disadvantages and Key Risks
Capillary underfill	Maximum reliability for demanding components	Flip-chip assemblies, especially on organic substrates	Highest reliability after curing, proven, and well understood	Slow process (bottleneck), voids, incomplete fill, not reworkable, and package warpage
No-flow underfill	Production speed and low cost	Streamlined flip-chip assembly for SMT production lines	Fast, SMT-compatible, and low manufacturing cost	High CTE (lower reliability, risk of defective solder joints, and high risk of voiding
Wafer-level underfill	Ultimate scalability and cost per unit	Wafer-level packaging for modern fine-pitch packages	Lowest cost per unit in mass production and best for fine pitch	Complex material science (B-stage), risk of filler entrapments, and difficult to rework
Molded underfill	Integration and miniaturization (SiP)	Mass-produced packages for mobile devices, Fan-Out, and 2.5D/3D packages	Integrates underfill and molding and allows for denser package design	High risk of under-chip voids, high initial equipment cost, and warpage are critical issues
Board-level underfill	Reliability in harsh environments and demanding applications	BGA/CSP components in harsh environments (automotive and aerospace) or subjected to shocks (portable applications)	Dramatically increases drop/vibration resistance and provides protection from external influence	Can reduce thermal cycling life (if material CTE is high), additional process step, and difficult to inspect and control quality (voids)
Edge/corner bonding	Cost-effective drop/shock resistance	BGA/CSP components in portable and consumer electronics to reduce cost and production time	Very low cost and fast application, significant drop test improvement, and easy rework	Can severely worsen thermal cycling life, only partial protection, and stress concentration at corners

## Data Availability

The work data are presented within the publication in figures, tables, and text. Thus, no information on dataset sharing is stated.

## Abbreviations

The following abbreviations are used in this manuscript:

BGA	Ball grid array
C4	Controlled collapsed chip connection
CSP	Chip-scale package
CTE	Coefficient of thermal expansion
CUF	Conventional/capillary underfill
EMC	Epoxy molding compound
FR	Flame retardant
h	Separation distance
IC	Integrated circuit
L	Flow distance
MUF	Molded underfill
NCF	Non-conductive film
NCP	Non-conductive paste
NUF	No-flow underfill
OBAR	Over-bump applied resin
PCB	Printed circuit board
PCUF	Partial capillary underfill
ppm	Parts per million
SAM	Scanning acoustic microscopy
SiP	System-in-package
SMT	Surface mount technology
t	Time
TC	Thermo-compression
T_g_	Glass transition temperature
WUF	Wafer-level underfill
γ	Surface tension
θ	Wetting angle
Μ	Absolute viscosity
